# A pH-Responsive DNA Tetrahedron/Methotrexate Drug Delivery System Used for Rheumatoid Arthritis Treatment

**DOI:** 10.3390/jfb14110541

**Published:** 2023-11-03

**Authors:** Yi Jin, Xingyu Ge, Yinjin Xu, Siyi Wang, Qian Lu, Aidong Deng, Jingjing Li, Zhifeng Gu

**Affiliations:** 1 Department of Rheumatology, Affiliated Hospital of Nantong University, Medical School of Nantong University, Nantong 226000, China; jin_yibai@126.com (Y.J.); 2 Research Center of Clinical Medicine, Affiliated Hospital of Nantong University, Nantong 226000, China; 3 Department of Rheumatology, Yancheng Third People’s Hospital, Yancheng 224000, China; 15896260521@163.com; 4 Department of Hand Surgery, Affiliated Hospital of Nantong University, Nantong 226000, China

**Keywords:** DNA tetrahedron, methotrexate, macrophage, Rheumatoid arthritis

## Abstract

Rheumatoid arthritis (RA) is a chronic autoimmune disorder that leads to progressive and aggressive joint inflammation. The disease process is characterized by the activation of macrophages, which then release tumor necrosis factor-α (TNF-α) and interleukin-1β (IL-1β), accelerating tissue damage. Tackling tissue damage is a crucial target in the treatment of RA. In this study, a macrophage-targeted and pH-response DNA tetrahedron/methotrexate drug delivery system was constructed by loading methotrexate (MTX) onto a DNA duplex. MTX was used as a drug model, and a pH-response DNA tetrahedron (TET) was used as the drug carrier, which was modified with hyaluronic acid (HA) to target macrophages. The aim of this study was to evaluate the potential of TET as an effective drug carrier for the treatment of RA. On this basis, we successfully prepared TETs loaded with MTX, and in vitro assays showed that the MTX-TET treatment could successfully target macrophages and induce macrophages to polarize to M1 phenotype. At the same time, we also injected MTX-TET intravenously into collagen-induced arthritis (CIA) model mice, and the redness and swelling of the paws of mice were significantly alleviated, proving that the MTX-TET could successfully target inflamed joints and release MTX to treat joint swelling. In addition, the histochemical results showed that the MTX-TET could reduce synovitis and joint swelling in CIA mice, reduce the level of inflammatory factors in vivo, and improve the disease status while maintaining a good biosafety profile. This study showed that the MTX-TET treatment has beneficial therapeutic effects on RA, providing a new strategy for the clinical treatment of RA.

## 1. Introduction

Rheumatoid arthritis (RA) is an autoimmune disease involving the joints [1], and it is characterized by progressive and aggressive behavior. The primary pathological changes associated with RA are synovial inflammation and the formation of vascular opacities [2]. Approximately half of RA patients develop varying degrees of bone destruction [3], with joint deformity in severe cases. Bone erosion and bone destruction are also common in RA. In recent years, with the continuous developments in cutting-edge treatment principles, advanced concepts such as early diagnosis and treatment, standardized treatment, and targeted therapy have been introduced one after another, and the prognoses for RA patients have improved significantly. However, the proportion of patients with bone erosion and bone destruction is still high, and some patients still experience joint deformity, dysfunction, and even loss, which ultimately leads to a significant decline in the quality of life of patients [4].

The drugs currently used in clinical practice can be divided into three categories: disease-modifying antirheumatic drugs (DMARDs), non-steroidal anti-inflammatory drugs (NSAIDs), and glucocorticoids (GCs) [5]. Methotrexate (MTX) is a commonly used drug in the treatment of RA, with good efficacy, tolerability, and safety compared to other disease-modifying antirheumatic drugs (DMARDs) [6]. MTX has been shown to promote the apoptosis of macrophages [7], inhibit TNF-α secretion, reduce the infiltration of macrophages in the joint synovium, and restore the synovial structure to normal [8]. However, when MTX is administered systemically, it is distributed throughout the body via blood circulation, making it difficult to target pathological tissues effectively [9]. Additionally, the systemic administration of MTX is associated with multiple adverse effects [10]. These limitations have prompted the search for new drug delivery carriers and strategies to improve drug bioavailability, reduce the side effects in the systemic circulation, and achieve better therapeutic outcomes.

The pathological development of RA is characterized by inflammatory damage that leads to the formation of a local hypoxic and acidic microenvironment in synovial tissues [11]. This is due to the large amounts of anaerobic glycolysis in the synovial fluid [12], which has a pH of approximately 6.6 in the inflammatory joint sites of RA patients, compared to 7.3 in normal joints [13]. In this regard, the strategy of using pH-sensitive nanocarriers bearing therapeutic drugs (e.g., NSAIDs, and GCs) has great potential in RA treatment. In recent years, pH-sensitive responsive nanomaterials have gained importance because of their limited side effects, minimal doses, and controlled drug release. pH-responsive polymer carriers can improve the efficiency of drug delivery in vivo, allow targeted drug delivery, and reduce adverse drug reactions, enabling multifunctional and personalized treatment [14]. A multi-functional FRβ-targeting and pH-responsive nanocarriers designed by Teng’s team [15] displayed pH-sensitive release at pH 5.0 in vitro and superior cellular uptake toward activated macrophages, which resulted in the greater inhibition of cell proliferation. After the pH-sensitive nanocarrier delivered the drug to the affected area, low-pH conditions triggered the precise release of the drug to its site of action. The application of this method could improve the efficacy of RA treatment by improving treatment selectivity and reducing systemic adverse reactions. Furthermore, under acidic conditions, drug delivery systems tend to demonstrate burst drug releases instead of extended releases. Therefore, engineering DNA as a good drug carrier has been widely used in the treatment and diagnosis of diseases. For example, DNA tetrahedrons (TETs) are stable and easy to synthesize and have a wide range of applications in the treatment of autoimmune diseases [16]. A pH-responsive TET nano platform enables the precise and effective loading of therapeutic drugs with controlled drug releases for the weakly acidic microenvironment of RA. In recent years, the use of TETs has gained considerable attention in the biomedical field due to its permeability to cell membranes, low cytotoxicity [17], and stability [18]. TETs have been used in various treatments for diseases, such as inserting Adriamycin into the GC-rich regions of TETs to combat drug resistance in tumor cells [19]. A nucleic acid tetrahedron framework loaded with baicalein was also synthesized and used to treat osteoarthritis. TETs linked with siRNA and modified with an aptamer that targets glioma cells have been found to solve the problem of siRNA in vivo [20,21]. These studies have provided further inspiration for the application of TETs in RA treatment.

Additionally, in RA, various immune cells, including macrophages, are activated and infiltrate the synovial membrane to contribute to synovial inflammation [22,23]. Macrophages play a crucial role in the pathogenesis of RA. Studies have identified two distinct polarization states of macrophages: classically activated macrophages (M1) and alternatively activated macrophages (M2) [24]. M1-type macrophages release inflammatory factors such as TNF-α and IL-1β [25] and promote inflammation, while M2-type macrophages produce anti-inflammatory factors (e.g., IL-10 and TGF-β) and contribute to tissue repair and remodeling [26]. The imbalanced ratio between M1 and M2 macrophages is a key factor in the pathogenesis of RA [27]. Research on the reprogramming of macrophages for RA treatment has been conducted, such as the use of M2 exosomes to alleviate RA by converting inflammatory M1-type macrophages into anti-inflammatory M2-type macrophages [28,29]. Therefore, macrophages could serve as a potential therapeutic target in the treatment of RA.

**Scheme 1:**
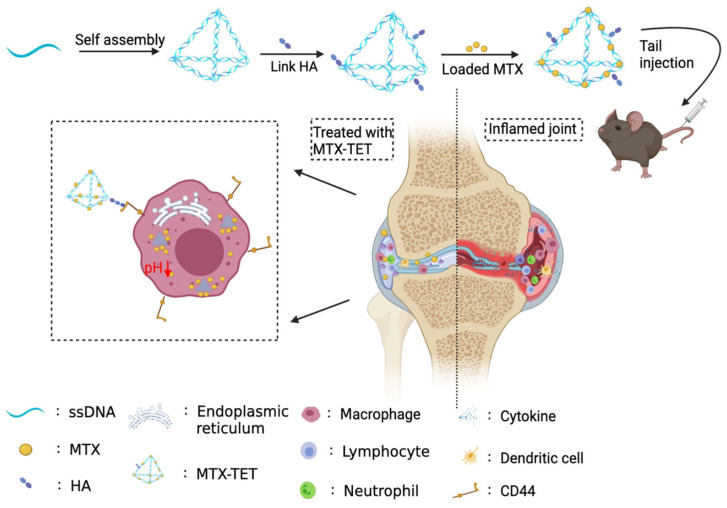
Schematic illustration of the targeted delivery of the MTX-TET treatment to an inflamed joint in RA.

In this study, we aimed to construct a methotrexate-loaded DNA tetrahedron (MTX-TET) to treat RA (Scheme 1). Hyaluronic acid (HA) possesses a special CD44 receptor binding ability, which can specifically target macrophages that have been conjugated to TET according to the NHS-EDC reaction [30]. HA-TET can target activated macrophages at the site of inflammation. We prepared an MTX-TET treatment and examined its properties, its effect on macrophage viability and polarization, and its therapeutic effect on CIA model mice. Our findings offer a new approach for the clinical treatment of RA.

## 2. Materials and Methods

### 2.1. Materials

Anti-mouse CD31, anti-mouse CD68, and anti-mouse CD86 antibodies were purchased from Abcam (Cambridge, UK). High glucose Dulbecco’s modified Eagle’s medium (DMEM), penicillin and streptomycin, trypsin, and fetal bovine serum (FBS) were purchased from Gibco (Grand Island, NY, USA). Lipopolysaccharide (LPS) was purchased from Sangon (Biotech, Shanghai, China). MTX was purchased from Selleck Chemicals (Houston, TX, USA). A cell viability detection kit (CCK-8) and DAPI were purchased from Beyotime (Shanghai, China). Freund’s complete adjuvant and Freund’s incomplete adjuvant were purchased from Sigma (Saint Louis, MO, USA). Bovine type II collagen was purchased from Chondrex (CFA, Chondrex, Woodinville, WA, USA). A first-strand cDNA synthesis kit and trizol reagent were purchased from Thermo Fisher (Waltham, MA, USA). The RAW264.7 cell line was purchased from the National Collection of Authenticated Cell Cultures (Shanghai, China).

### 2.2. Preparation and Characterization of MTX-TET

As shown in Scheme 1, S1/S1M, S2, S3, and S4 (Table 1) were added in equal proportions to TM buffer (10 mmol/L Tris-HCl and 50 mmol/L MgCl_2_, pH 8.0), which was then synthesized using a PCR system at 95 °C for 10 min before being returned to room temperature gradually. The conjugation between the carboxylate groups of HA and the ammonium groups of TET was performed according to the NHS-EDC reaction [31]. The detail was as follows: 0.2 mM HA was bound to S1M in the synthesized TET using the NHS-EDC reaction system (containing 0.4 mg/mL EDC and 0.6 mg/mL NHS) at room temperature for 15 min and terminated with 2-Mercaptoethanol. The preparation of the MTX-TET treatment was performed according to the reported method [32,33]. Specifically, a mixture of 10 μM TET and 10 μM MTX was incubated for 12 h, added to an ultrafiltration tube (10 kD), and centrifuged at 4000 rpm for 20 min at 4 °C. The filtrate from the outer tube was taken to obtain MTX-TET. PAGE electrophoresis (Bio-Rad, Irvine, CA, USA) was used to certify the successful construction of the TET. In addition, nanoparticle tracking analysis (NTA) was established to detect the size and potential of the TET (Particle Metrix, Meerbusch, Germany). Spectrophotometry (Thermo Fisher Scientific, Waltham, MA, USA) was utilized to calculate the loading efficiency of the MTX in the TET.

### 2.3. Cell Culture

The RAW264.7 cell line was cultured in DMEM, and the culture media was supplemented with 10% FBS and 1% antibiotic (100 mg/mL streptomycin and 100 U/mL penicillin). The cells were cultured in a 37 °C thermostatic incubator with an atmosphere of 5% CO_2_. The round glass slides, after pickling and sterilization, were put into a 24-well plate, and the RAW264.7 cells suspension was adjusted to a concentration of 1 × 10^5^ cells/well and spread on the 24-well plates for 24 h. Afterward, 10 μM FITC modified TET (FITC-HA-TET) was co-cultured with RAW264.7 cells for 6 h. After rinsing with PBS, the macrophages were fixed with 4% paraformaldehyde (PFA) and stained with DAPI. The slides were observed under a fluorescence microscope (Olympus, Tokyo, Japan).

### 2.4. qRT-PCR Analysis

Firstly, trizol was applied to extract the total RNA from the RAW264.7 cells. Then, RNA was reverse-transcribed into cDNA with a First-strand cDNA synthesis kit (Thermo Fisher Scientific, Waltham, MA, USA) and quantified using SYBR^®^ Green PCR master mix with Quanti-Studio 5 (Applied Biosystems, Foster City, CA, USA). The expression of the target genes was calculated using the 2–DDCt comparative method for relative quantification after normalization against glyceraldehyde 3-phosphate dehydrogenase (GAPDH). To detect IL-6 and iNOS RNA expression, the following primers were used: IL-6 forward 5′-CTGCAAGAGACTTCCATCCAG-3′; IL-6 reverse 5′-AGTGGTATAGACAGGTCTGTTGG-3′; iNOS forward 5′-GTTCTCAGCCCAACAATACAAGA-3′; and iNOS reverse 5′-GTGGACGGGTCGATGTCAC-3′.

### 2.5. Establishment and Treatment of the Collagen-Induced Arthritis Model

All animal procedures were performed according to the experimental protocol approved by the Animal Ethics Committee of Nantong University. The DBA/1 mice were randomly divided into the following five groups: a normal group, a saline group, a TET group, an MTX group, and an MTX-TET group. Bovine II type collagen was mixed with complete Freund’s adjuvant in equal proportions to create the final concentration of 1 mg/mL, and 0.1 mL of the mixture was subcutaneously injected into the tail base of each mouse. One week later, their immune systems were re-immunized with the mixture. After model establishment, each mouse in the normal group, the saline group, the TET group, the MTX group, and the MTX-TET group was injected with saline (0.1 mL), TET (40 μM, 0.1 mL), MTX (10 μM, 0.1 mL), and MTX-TET (40 μM, 0.1 mL), respectively, once per week for a total of three weeks.

### 2.6. Clinical Assessment of Arthritis

After secondary immunity, according to the following standards for each limb on a 4-point scale, was assessed the mice according to the following criteria: 0 = normal foot claws; 1 = erythema and mild swelling limited to the tarsi or ankle joints; 2 = erythema and mild swelling extending from the ankle to the tarsus; 3 = erythema and moderate swelling extending from the ankle to the metatarsal joint; and 4 = erythematous and severely swollen ankles, feet, and digits, or ankylosed limbs [34]. The scores of the four feet were summed to obtain an arthritis score.

### 2.7. Flow Cytometry

The RAW264.7 cells were collected and dispersed in a staining buffer. The surface proteins were stained with FITC-CD86, PE-F4/80, and APC-CD206 antibodies for half an hour under four-degree conditions, and they were washed with PBS three times. Finally, the suspension was analyzed with a BD FACS Calibur (BD, Franklin Lakes, NJ, USA). The data analysis used Flowjo.v10 software.

### 2.8. Immunohistochemical and Histology Analysis

Synovium tissue sections were decalcified in a 10% ethylenediamine tetraacetic acid (EDTA) solution for 1 month, with the solution being changed every 2 days. The samples were then dehydrated and embedded in paraffin. The tissue sections were cut into 5 μm thickness with a microtome. After dewaxing and rehydrating, the sections were subjected to immunohistochemical analysis with anti-mouse CD31, anti-mouse CD68, and anti-mouse CD86 antibodies. Additionally, the main organs, including the heart, liver, spleen, lung, and kidney were harvested, and after being fixed in 4% paraformaldehyde for 24 h, after which, they were embedded in paraffin, sectioned at 3–5 µm, and stained with hematoxylin and eosin (HE) for pathological analysis.

### 2.9. Micro-CT Analysis

The hard tissues, including the paws, ankle joints, and distal tibia, were fixed in 4% formalin overnight and analyzed using a Micro-CT system (Juli, Guangzhou, China). The 3D-reconstructed models and sectional images were created using CTVox 3.3 and Data Viewer software v4.1.1. To evaluate the bone-related parameters, the area surrounding the distal tibia was selected as the region of interest (ROI), where the bone volume (BV), tissue volume (TV), BV/TV ratio, and Tb. Th were determined.

### 2.10. Enzyme-Linked Immunosorbent Assay (ELISA)

The concentrations of TNF-a, interleukin (IL)-10, and RF (R&D Systems, Minneapolis, MN, USA) in the mouse serum samples were measured using ELISA kits according to the manufacturer’s instructions. The absorbance was read at 450 nm using a microplate reader.

### 2.11. Statistical Analysis

All data are representative of the results from at least three independent experiments. The results are shown as means ± SDs and the *t*-tests were used to determine the statistical significance. All statistical analyses were performed with GraphPad Prism9.

## 3. Results

### 3.1. Characterization of MTX@TET@HA Nanoparticles

**Figure 1:**
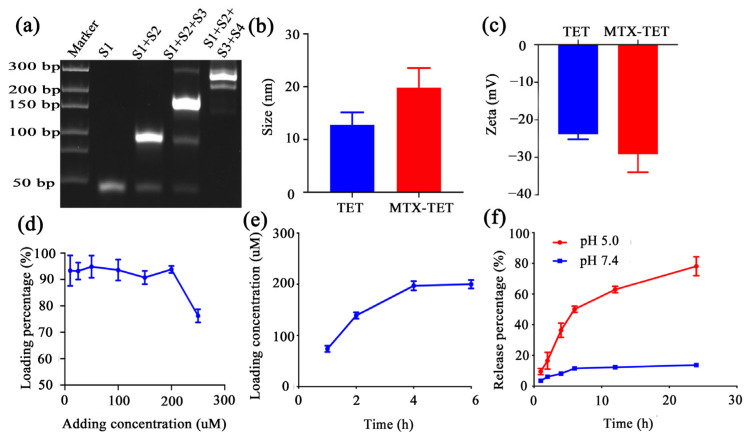
Characterization of the MTX-TET nanoparticles. (**a**) PAGE electrophoresis of the TET. (**b**) The particle sizes of the TET and MTX-TET. (**c**) The zeta potentials of the TET and MTX-TET. (**d**) The loading efficiency of the MTX in the TET. (**e**) The minimum loading time. (**f**) The release efficiency of the MTX under different pH values.

The formation of the TETs was characterized using polyacrylamide gel electrophoresis (PAGE) analysis. The result showed that the migration rate of bands S1 plus S2 plus S3 plus S4 was lower than that of S1, S1 plus S2, and S1 plus S2 plus S3, as shown in Figure 1a. The particle size and zeta potential of the TET were determined using a zeta viewer, and they were found to be 15.89 nm and −23.2 mV, respectively. After loading the MTX, the particle size increased to 36.11 nm, and the zeta potential was decreased to −28.75 mV (Figure 1b,c). These results showed the successful connection of the MTX. MTX has the maximum absorbance at 340 nm [35]. To detect the payload of the MTX in the TET, we detected the OD value of 340 nm through the enzyme-labeled instrument. According to the data analysis, the loading efficiency of the MTX in the TET was optimized. When the concentration of the TET was fixed at 10 μM, the amount of MTX loaded on the TET increased with the rising amount of added MTX, and the saturation concentration was 190.6 μM (Figure 1d). Similarly, when the TET was fixed at 10 μM, and the added MTX concentration was 250 μM, the required saturation time was 4 h (Figure 1e).

**Figure 2:**
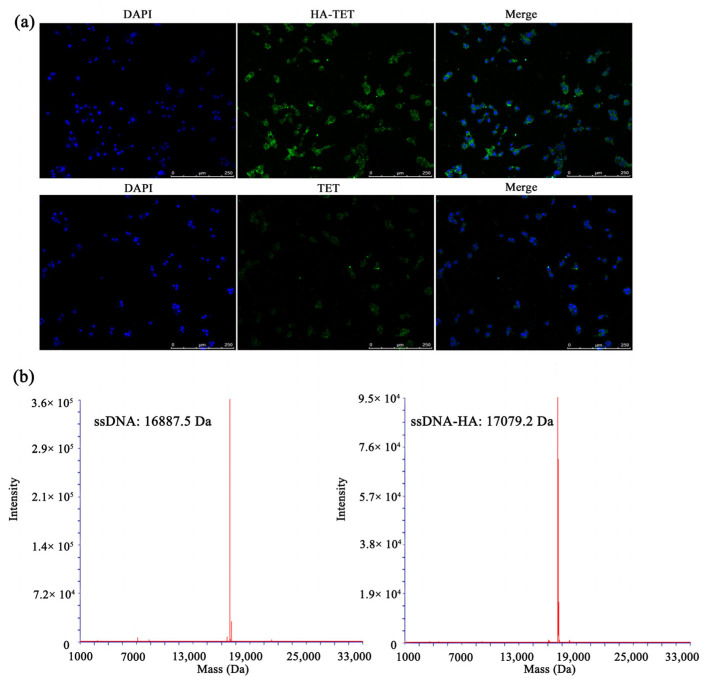
Characterization of the HA-TET/ssDNA-HA nanoparticles. (**a**) Representative fluorescence image of the TET uptake by the RAW264.7 cells; (scale bar: 250 μm). (**b**) Mass spectrometry analysis of the ssDNA and the ssDNA-HA.

Finally, the MTX-TET was incubated in PBS solution of different pH values. The results showed that at a pH of 5.0, 83.4% of the MTX was released after 24 h of incubation, while only 14.0% was released at a pH of 7.4 (Figure 1f). However, the concentrations of the purified TET and HA-TET were determined by measuring the absorbance at 260 nm under different pH values, and the results showed stability in the acidic environments (Figure S1c). This indicated that the MTX-TET was relatively stable in blood circulation, but it quickly released MTX in the acid microenvironment of RA. When the HA-TET was co-cultured with macrophages, the immunofluorescence results showed that the HA-TET could bind to the macrophages (Figure 2a). The attachment of the HA to the ssDNA was confirmed by mass spectrometry, with a molecular weight that changed from 16,887.5 Da to 17,079.2 Da, indicating the successful attachment (Figure 2b). The particle size and zeta potential of the HA-TET were determined using a zeta viewer, and the results showed the successful linkage of the HA to the TET (Figure S1a,b).

### 3.2. MTX-TET Could Affect the Polarization of the RAW264.7 Cells

**Figure 3:**
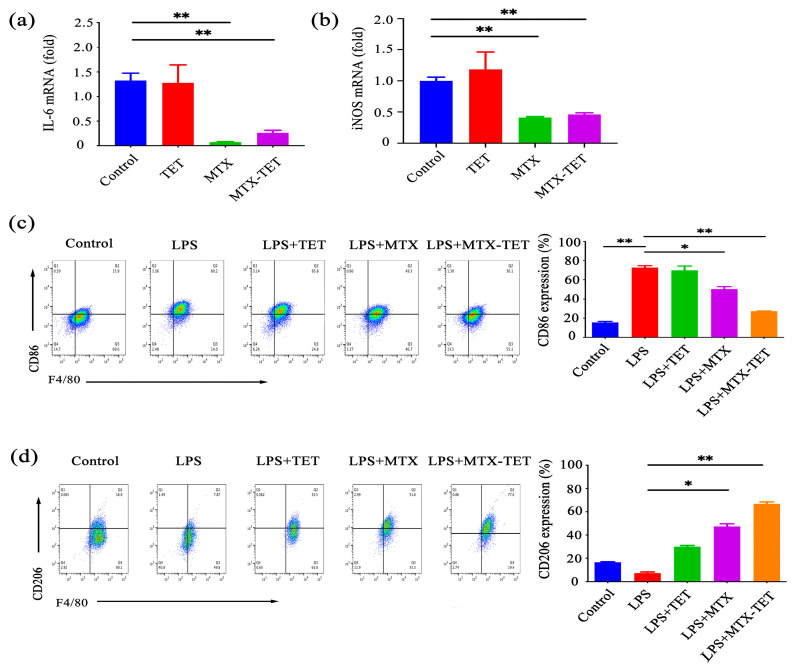
The characterization of the macrophage polarization induced by the MTX-TET. (**a**,**b**) PCR showing the expression of the pro-inflammatory genes IL-6 (**a**) and iNOS (**b**) in the RAW264.7 cells. (**c**,**d**) Flow cytometry showing the expression of the pro-inflammatory phenotype marker CD86 (**c**) and anti-inflammatory phenotype marker CD206 (**d**). (*n* = 5. * *p* < 0.05, ** *p* < 0.01).

To further assess the impact of the MTX-TET on the phenotypes of the macrophages, we stimulated the RAW264.7 cells with LPS to induce differentiation towards the M1 phenotype, dividing the cells into four groups. The M1 phenotype was then stimulated with base culture-medium, TET, 10 μM MTX, and 10 μM MTX-TET. The qRT-PCR assays demonstrated that MTX significantly reduced the expression of the inflammation-related genes IL-6 and iNOS mRNA in the RAW264.7 cells, and MTX-TET significantly reduced the expression of iNOS mRNA. The expression of IL-6 in the MTX-TET group was also reduced compared to the TET group (Figure 3a,b). Flow cytometry (FCM) showed that the MTX-TET significantly decreased the expression of the M1 phenotype-associated marker CD86 and increased the expression of the M2 phenotype-associated marker CD206 in the RAW264.7 cells (Figure 3c,d). The experiments suggested that the MTX-TET could inhibit the polarization of the RAW264.7 cells towards the pro-inflammatory M1 phenotype.

### 3.3. RA-Targeting Ability of the MTX-TET

**Figure 4:**
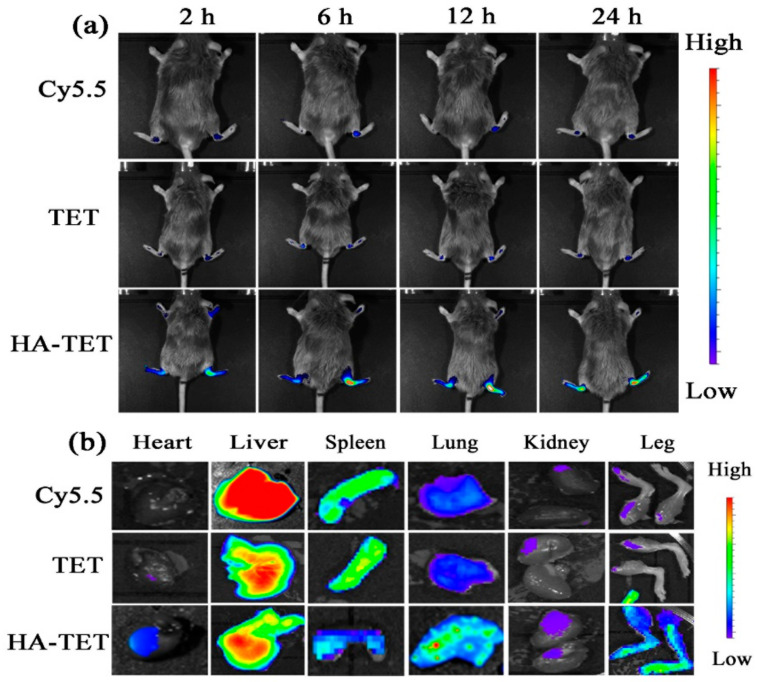
Living image of the CIA mice. (**a**) The overall view. (**b**) The image of each organ, such as the heart, liver, spleen, lung, kidney, and leg; (*n* = 3).

To assess the specific targeting of the HA-TET to inflamed tissues in RA, Cy5.5 solution, a TET solution, and HA-TET were injected intravenously into the CIA mice and the fluorescence signals of their hind paws were monitored using a near-infrared imaging system (NIRF). Real-time imaging showed that the fluorescence intensity reached its maximum value at 12 h, and the fluorescence intensity of the HA-TET group was much higher than the TET and Cy 5.5 groups, indicating that the HA-TET could accumulate in the inflamed joint (Figure 4a). Further analysis of the biodistribution of the TET and HA-TET in major organs and inflamed paws 24 h after injection revealed that the TET and HA-TET were predominantly distributed in the inflamed paws, and the fluorescent signals in the HA-TET group were much stronger than that of the TET group (Figure 4b). These results suggested that the targeting efficiency of the HA-TET was significantly improved compared to that of the TET alone.

### 3.4. The Curative Effect of MTX-TET

**Figure 5:**
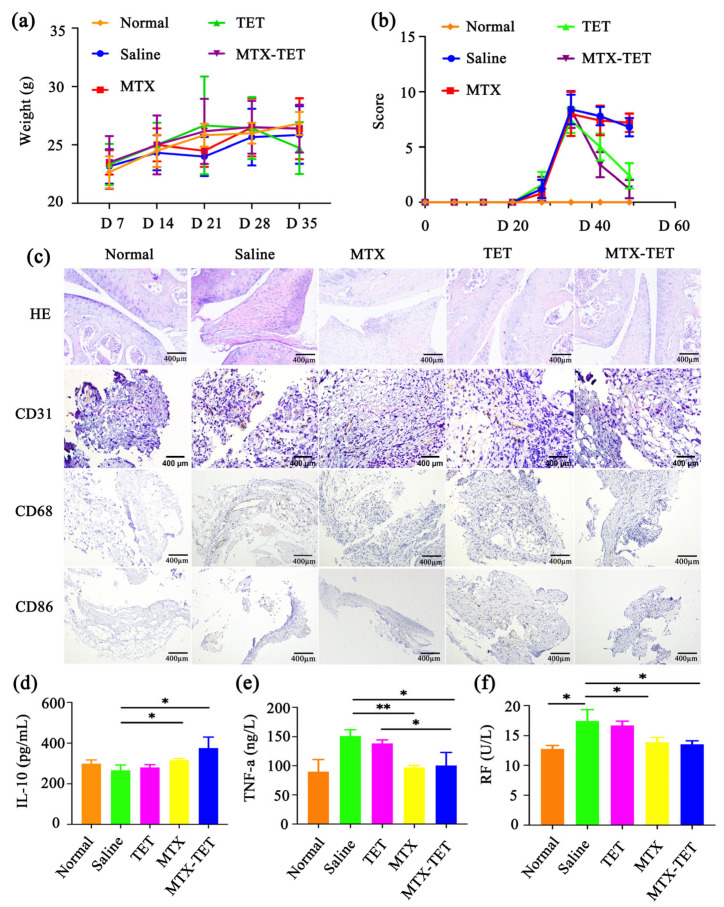
The therapeutic effect of the TET in the CIA mice. (**a**) The score of each group; (*n* = 5). (**b**) The weight of each group; (*n* = 5). (**c**) Representative photo of the IHC of each group during the treatment, including the pathological change in the HE-stained synovium samples; (CD31 immunohistochemistry, CD68 immunohistochemistry, and CD86 immunohistochemistry). (scale bar: 400 μm). (**d**–**f**) The concentration of the inflammatory factors: IL-10 (**d**), TNF-α (**e**), and RF (**f**); (*n* = 5; * *p* < 0.05, ** *p* < 0.01).

**Figure 6:**
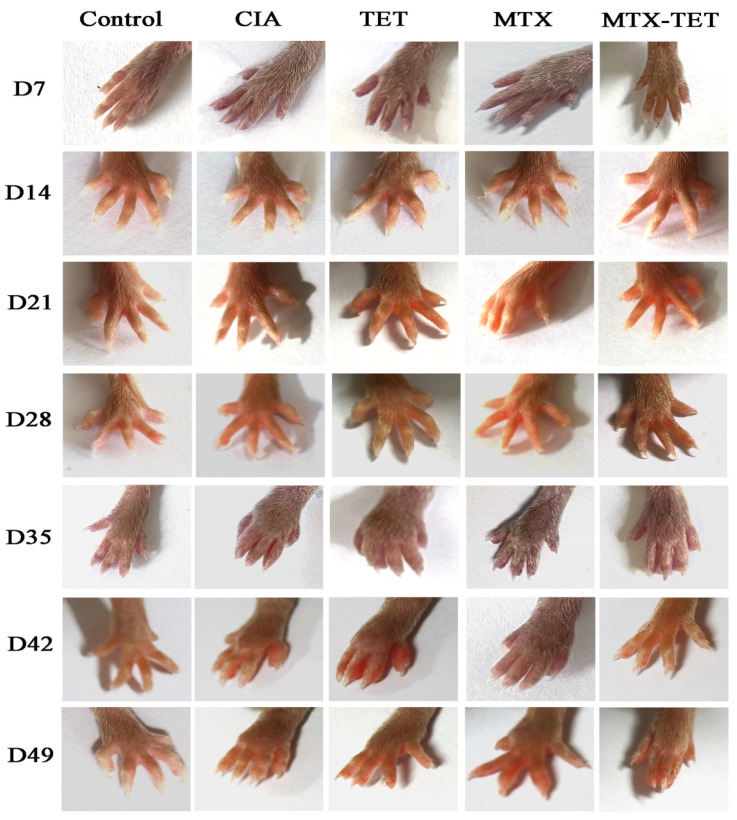
Representative images of the paws in each group.

The CIA model mice were randomly divided into normal, saline, TET, MTX, and MTX-TET groups. All groups except the normal group were modeled and immunized twice on day zero and day seven, respectively. Weekly observations, weighings, and evaluations of the CIA modeling scores were conducted. The results showed that the MTX-TET could significantly reduce the degree of joint swelling, with a negligible weight change (Figure 5a). Additionally, the CIA modeling scores for the MTX and MTX-TET groups were obviously decreased, with the alteration being higher in the MTX-TET group (Figure 5b). Compared to normal mice, the CIA mice treated with the MTX-TET showed relief of disease symptoms, including reduced paw redness and swelling (Figure 6). After three weeks of treatment, the mice were sacrificed, and their joint synovium samples were collected for examination. The synovium samples were embedded in paraffin sections for HE-staining and immunohistochemical (IHC) staining using CD31, CD68, and CD86 antibodies. The HE-staining results revealed an even distribution and regular arrangement of the synovial cells in the normal group. On the other hand, the cells in the saline and TET groups exhibited abnormal proliferation and a substantial number of infiltrated inflammatory cells. The MTX-TET group showed improvements in synovial hyperplasia, with less infiltration of inflammatory cells (Figure 5c). CD31 is commonly used to demonstrate the presence of endothelial cells in tissues and to assess vascular density in tissues [36]. The IHC results showed that the expression of CD31 in the synovial membranes of the saline group was significantly higher compared to that of the normal group, while it was reduced in the MTX group and not present in the MTX-TET group (Figure 5c). The expression of CD68, a 110 kD glycoprotein expressed by macrophages and associated with lysosomes [37], was higher in the saline group than it was in the normal group, but after the MTX-TET treatment, the inflammation was dramatically reduced (Figure 5c). Meanwhile, CD86 is considered a reliable biomarker for disease severity and response to therapy [38]. The expression of CD86, a typical marker of macrophage M1, was decreased in the MTX-TET group (Figure 5c). Therefore, the MTX-TET treatment reduced the inflammation and prevented damage to the bones and cartilage tissues in the ankle joints of the CIA mice.

Furthermore, the levels of inflammatory factors in the serum samples were measured by ELISA. The results showed a significant increase in the expression of the anti-inflammatory factor IL-10 (Figure 5d), while the levels of the pro-inflammatory factor TNF-α (Figure 5e) decreased significantly in the MTX-TET group. After a period of treatment, the rheumatoid factor (RF) levels in the mice were significantly reduced, and the extent of the disease was alleviated (Figure 5f).

### 3.5. Microcomputed Tomography Analysis

**Figure 7:**
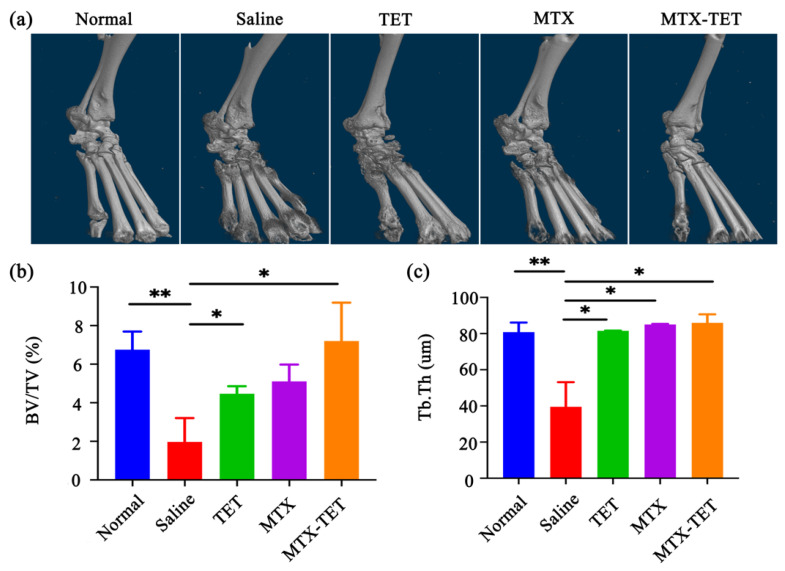
Micro-CT scans of the CIA mice. (**a**) 3D image reconstruction. (**b**,**c**) The ratios of the bone volume fractions (BV/TV) and the trabecular thickness of bone (Tb. Th); (*n* = 5. * *p* < 0.05, ** *p* < 0.01).

Finally, we retained the distal tibia of the right hind limbs, ankle joints, and paws of the mice, and we performed a micro-CT scan and 3D reconstruction after removing the excess soft tissues (Figure 7a). The results indicated that the CIA group had obvious bone damage in the ankle and toe joints. The TET did not show a significant therapeutic effect. The free MTX showed good results in treating the CIA mice, but slight bone destruction was still present. In contrast, the MTX-TET group showed the significant alleviation of bone destruction. The data analysis revealed that the MTX-TET could significantly improve the ratios of the bone volume fractions (BV/TV) and the trabecular thickness of bone (Tb. Th) (Figure 7b,c).

### 3.6. The Safety of the MTX-TET

**Figure 8:**
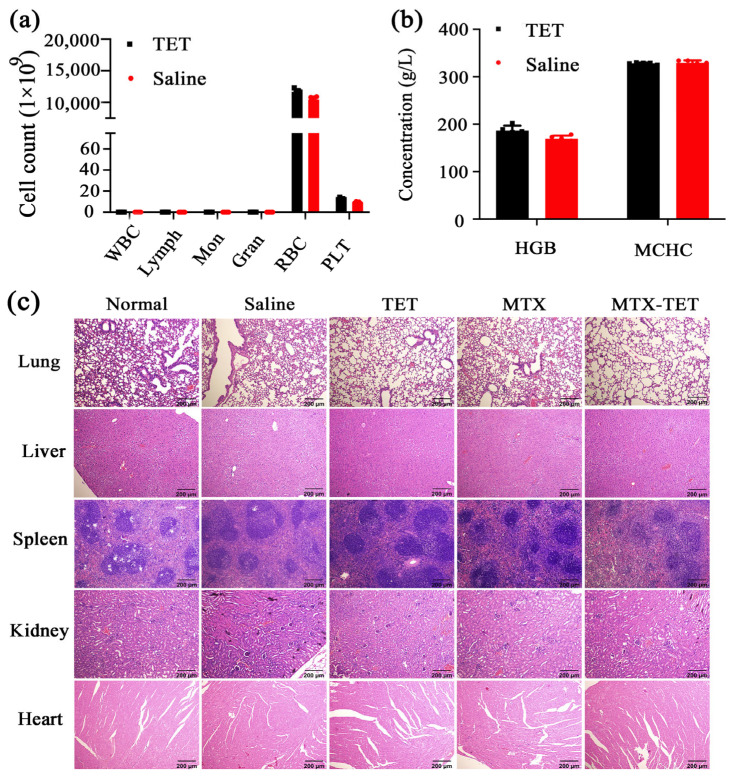
The safety of the TET. (**a**) Cell counts (WBC, Lymph, Mon, Gran, RBC, and PLT) in the TET-treated group and the PBS-treated group. (**b**) Blood routine examination parameters after TET treatment. (**c**) HE-staining of the organs (the lungs; livers; spleens; kidneys and hearts). (*n* = 5; scale bar: 200 μm).

To evaluate the safety of the MTX-TET, a thorough examination of the mice’s blood samples was conducted. The results, depicted in Figure 8a, demonstrated that there were no differences in the proportions of white blood cells (WBC), including lymphocytes (Lymph), monocytes (Mon), and granulocytes (Gran), as well as the red blood cells (RBC), and platelets (PLT), between the MTX-TET and control groups. Moreover, the function of the red blood cells was evaluated through the indices of hemoglobin (HGB) and mean corpuscular hemoglobin concentration (MCHC). The results, shown in Figure 8b, indicated that the levels in the TET-treated group were comparable to those in the saline-treated group. The results of the HE-staining for the major organs such as the lungs, livers, spleens, kidneys, and hearts showed that there were no obvious lesions in any of the organs of the MTX-TET groups (Figure 8c). These results showed the good biocompatibility of the TET.

## 4. Discussion

In this study, we successfully prepared a TET loaded with MTX, verified its effect on RAW polarization, and preliminarily explored its application in the treatment of CIA model mice. Our study showed that the TET, as an efficient and safe drug delivery carrier, has good application prospects for the treatment of RA, and it has the potential to be applied to clinical treatment. Precise delivery of the TET to inflamed joints as a pH-responsive delivery vehicle can significantly improve the therapeutic index of antiradicals. pH-responsive polymer systems are a class of materials that can change their physicochemical properties in response to changes in pH levels [39].

In this study, a method from previous research was replicated, where four ssDNA samples were mixed in equal proportions, and then briefly heated to 95 °C and annealed at 4 °C. The complementary bases within each ssDNA autonomously paired and assembled into a tetrahedral structure. PAGE gel electrophoresis revealed that the ssDNA migrated to the lowest position, whereas the TET migrated to the highest, confirming the successful synthesis of the TET. The TET, made up of nucleic acids, boasted a sturdy geometrical structure, endowing it with remarkable modifiability. The amino group (-NH2) on the ssDNA sequence was modified and subsequently linked to HA using the EDC-NHS cross-linking activation method. Mass spectrometry confirmed the successful attachment of the HA to the ssDNA. Given that HA is a specific ligand for the CD44 molecule, which is abundantly expressed on the surfaces of activated RAW264.7 cells, the HA-modified TET demonstrated targeted interactions with these cells. When the FITC-HA-TET was co-cultured with the RAW264.7 cells, cellular immunofluorescence assays verified its internalization by the cells.

TETs are recognized as good drug carriers. Their double-stranded structures furnish numerous sites for MTX loading. MTX has the ability to intercalate into the duplex helix’s groove. During an incubation of MTX with the TET, an observed increase in particle size and potential changes indicated successful MTX-loading. TETs release their drug payloads in acidic environments, but they remain stable in neutral pH conditions. At an acidic pH of 5.0, the MTX displayed a rapid release rate of 83.4%. In contrast, at a neutral pH of 7.4, its release was more gradual, with a rate of just 14.0%. These observations suggested that the MTX-TET responded to acidic conditions and maintained stability during blood circulation. In an inflammatory joint’s microenvironment, this facilitates the swift release of MTX, enabling its therapeutic effects. Overall, the TET’s preparation proves to be straightforward and efficient. Its favorable interaction with the RAW264.7 cells, combined with its superior MTX-loading and acid responsiveness, underscored its potential as a stable and effective drug delivery vehicle.

Macrophages serve as crucial targets in mitigating synovial inflammation and ameliorating RA. In our investigations, inflammation-related gene expressions were as-sessed using PCR, while RAW264.7 polarization-related markers were detected via FCM. This analysis revealed that the MTX-TET has the potential to suppress the pro-inflammatory M1 phenotype of the RAW264.7 cells and foster the anti-inflammatory M2 phenotype.

Further in vivo examination of the MTX-TET’s effects demonstrated its capacity to pinpoint inflammatory joint sites and render therapeutic benefits. In vivo imaging showed that the MTX-TET could successfully target inflammatory joint sites and exert therapeutic effects. After a period of treatment, notable reductions in swelling were observed in the feet and paws of the mice treated with both MTX and the MTX-TET, with the latter group showing pronounced improvements. Given RA’s progressive nature with synovitis as a principal pathological manifestation, HE-staining was applied to the synovial pathological sections of the mouse groups. The findings showed marked reductions in synovial dysplasia, infiltrating inflammatory cells, and overall synovial inflammation. CD31, a standard marker for endothelial cell presence and vascular density assessment in tissues, was employed in an IHC staining procedure to assess the propensity for synovial pannus formation in mice. The data indicated pronounced reductions in the CD31 expression levels within the synovial membranes of the MTX-TET- treated mice compared to the CIA group, suggesting inhibited vascular proliferation. Moreover, declines in the serum inflammatory factor levels in the MTX-TET group vouched for the treatment’s efficacy. Micro CT scans of the mice’s ankles and subsequent 3D reconstructions revealed a significant abatement in bone destruction for the MTX-TET group. Conclusively, these findings validate MTX-TET’s efficacy in substantially reducing synovitis and bone destruction in CIA mice, enhancing their overall health status.

Nowadays, many existing stimulus-response systems lack degradability or biocompatibility, which significantly reduces their chances of reaching clinical applications. TET, as a biocompatible nanocarriers, can be safely eliminated to prevent their accumulation in the body, which is extremely important, and their pH-sensitive properties can better control drug release. The HE-staining of the paraffin sections of the various mice organs showed no obvious pathological changes in the organs of the mice in the TET and MTX-TET groups. The above results indicated that the TETs had good biological safety.

In the research field of nanocarriers, the trend of interdisciplinary integration such as chemistry, materials science, biochemistry, and immunology is gradually strengthening. pH-responsive nanocarriers have shown unique advantages and potential application prospects in RA diagnosis and treatment. Good biocompatibility, intelligent targeting, ultra-high loading rates, and intelligent drug release are still the main research topics of pH-responsive nanocarriers. At the same time, the difficulties in their complex structural design and synthesis amplification also have hindered the conversion of pH-responsive systems to industrial scale. In this regard, the easy preparation, easy modification, and good biocompatibility of TETs that are of great significance for further industrial conversion. We believe that this pH-responsive DNA nanoplatform will open up new avenues for the targeted treatment of RA.

## Figures and Tables

**Table 1:** The sequence of each ssDNA strand used in this work.

DNA	Sequence
Strand 1	ACA TTC CTA AGT CTG AA AC ATT ACA GCT TGC TAC AC GA GAA GAG CCG CCA TAG TA
Strand 2	TAT CAC CAG GCA GTT GA CA GTG TAG CAA GCT GTA AT AG ATG CGA GGG TCC AAT AC
Strand 3	TCA ACT GCC TGG TGA TA AA ACG ACA CTA CGT GGG AA TC TAC TAT GGC GGC TCT TC
Strand 4	TTC AGA CTT AGG AAT GT GC TTC CCA CGT AGT GTC GT TT GTA TTG GAC CCT CGC AT
Strand 1M	ACA TTC CTA AGT CTG AA AC ATT ACA GCT TGC TAC AC GA GAA GAG CCG CCA TAG TA-NH2

## Data Availability

All the participants were informed about the study and signed an informed consent form.

## Supplementary Materials

The following supporting information can be downloaded at: https://www.mdpi.com/article/10.3390/jfb14110541/s1, Figure S1: Characterization of the HE-TET nanoparticles. (a) The particle sizes of the TET and HA-TET. (b) The zeta potentials of the TET and HA-TET. (c) The OD values of the TET and HA-TET under different pH conditions.

## References

[1] Coutant F., Miossec P. (2020). Evolving concepts of the pathogenesis of rheumatoid arthritis with focus on the early and late stages. Curr. Opin. Rheumatol..

[2] Aletaha D., Smolen J.S. (2018). Diagnosis and Management of Rheumatoid Arthritis: A Review. JAMA.

[3] Niu Q., Gao J., Wang L., Liu J., Zhang L. (2022). Regulation of differentiation and generation of osteoclasts in rheumatoid arthritis. Front. Immunol..

[4] Weyand C.M., Goronzy J.J. (2021). The immunology of rheumatoid arthritis. Nat. Immunol..

[5] Caporali R., Germinario S., Kacsándi D., Choy E., Szekanecz Z. (2023). Start RA treatment–Biologics or JAK-inhibitors?. Autoimmun. Rev..

[6] Yu J., Zhou P. (2020). The advances of methotrexate resistance in rheumatoid arthritis. Inflammopharmacology.

[7] Pandey S., Rai N., Mahtab A., Mittal D., Ahmad F.J., Sandal N., Neupane Y.R., Verma A.K., Talegaonkar S. (2021). Hyaluronate-functionalized hydroxyapatite nanoparticles laden with methotrexate and teriflunomide for the treatment of rheumatoid arthritis. Int. J. Biol. Macromol..

[8] Wei K., Jiang P., Zhao J., Jin Y., Zhang R., Chang C., Xu L., Xu L., Shi Y., Guo S. (2022). Biomarkers to Predict DMARDs Efficacy and Adverse Effect in Rheumatoid Arthritis. Front. Immunol..

[9] Tian J., Chen T., Huang B., Liu Y., Wang C., Cui Z., Xu H., Li Q., Zhang W., Liang Q. (2023). Inflammation specific environment activated methotrexate-loaded nanomedicine to treat rheumatoid arthritis by immune environment reconstruction. Acta Biomater..

[10] Wang W., Zhou H., Liu L. (2018). Side effects of methotrexate therapy for rheumatoid arthritis: A systematic review. Eur. J. Med. Chem..

[11] Ke J., Zhang H., Bu Y., Gan P., Chen F., Dong X., Wang Y., Wu H. (2022). Metabonomic analysis of abnormal sphingolipid metabolism in rheumatoid arthritis synovial fibroblasts in hypoxia microenvironment and intervention of geniposide. Front. Pharmacol..

[12] Fearon U., Hanlon M., Floudas A., Veale D. (2022). Cellular metabolic adaptations in rheumatoid arthritis and their therapeutic implications. Nat. Rev. Rheumatol..

[13] Fukamachi T., Wang X., Mochizuki Y., Maruyama C., Saito H., Kobayashi H. (2013). Acidic environments enhance the inhibitory effect of statins on proliferation of synovial cells. Int. Immunopharmacol..

[14] Chu S., Shi X., Tian Y., Gao F. (2022). pH-Responsive Polymer Nanomaterials for Tumor Therapy. Front. Oncol..

[15] Zhao J., Zhao M., Yu C., Zhang X., Liu J., Cheng X., Lee R.J., Sun F., Teng L., Li Y. (2017). Multifunctional folate receptor-targeting and pH-responsive nanocarriers loaded with methotrexate for treatment of rheumatoid arthritis. Int. J. Nanomed..

[16] Hu Y., Chen Z., Zhang H., Li M., Hou Z., Luo X., Xue X. (2017). Development of DNA tetrahedron-based drug delivery system. Drug Deliv..

[17] Henry S.J.W., Stephanopoulos N. (2021). Functionalizing DNA nanostructures for therapeutic applications. Wiley Interdiscip. Rev. Nanomed. Nanobiotechnol..

[18] Hu Q., Li H., Wang L., Gu H., Fan C. (2019). DNA Nanotechnology-Enabled Drug Delivery Systems. Chem. Rev..

[19] Kim K., Kim D., Lee T., Yhee J., Kim B., Kwon I., Ahn D.J.C.c. (2013). Drug delivery by a self-assembled DNA tetrahedron for overcoming drug resistance in breast cancer cells. Chem. Commun..

[20] Zhou Y., Yang Q., Wang F., Zhou Z., Xu J., Cheng S., Cheng Y.J.I.j.o.n. (2021). Self-Assembled DNA Nanostructure as a Carrier for Targeted siRNA Delivery in Glioma Cells. Int. J. Nanomed..

[21] Xue H., Ding F., Zhang J., Guo Y., Gao X., Feng J., Zhu X., Zhang C. (2019). DNA tetrahedron-based nanogels for siRNA delivery and gene silencing. Chem. Commun..

[22] Siouti E., Andreakos E. (2019). The many facets of macrophages in rheumatoid arthritis. Biochem. Pharmacol..

[23] Tenshin H., Teramachi J., Ashtar M., Hiasa M., Inoue Y., Oda A., Tanimoto K., Shimizu S., Higa Y., Harada T. (2022). TGF-β-activated kinase-1 inhibitor LL-Z1640-2 reduces joint inflammation and bone destruction in mouse models of rheumatoid arthritis by inhibiting NLRP3 inflammasome, TACE, TNF-α and RANKL expression. Clin. Transl. Immunol..

[24] Zhao C., Song W., Ma J., Wang N. (2022). Macrophage-derived hybrid exosome-mimic nanovesicles loaded with black phosphorus for multimodal rheumatoid arthritis therapy. Biomater. Sci..

[25] Sly L.M., McKay D.M. (2022). Macrophage immunotherapy: Overcoming impediments to realize promise. Trends Immunol..

[26] Yunna C., Mengru H., Lei W., Weidong C. (2020). Macrophage M1/M2 polarization. Eur. J. Pharmacol..

[27] Wang Y., Han C.C., Cui D., Li Y., Ma Y., Wei W. (2017). Is macrophage polarization important in rheumatoid arthritis?. Int. Immunopharmacol..

[28] Kim H., Back J.H., Han G., Lee S.J., Park Y.E., Gu M.B., Yang Y., Lee J.E., Kim S.H. (2022). Extracellular vesicle-guided in situ reprogramming of synovial macrophages for the treatment of rheumatoid arthritis. Biomaterials.

[29] Han X., Li Q., Zhang S., Sun L., Liu W., Wang J. (2023). Inhibition of NEMO alleviates arthritis by blocking the M1 macrophage polarization. Int. Immunopharmacol..

[30] De Paula M.C., Carvalho S.G., Silvestre A.L.P., Dos Santos A.M., Meneguin A.B., Chorilli M. (2023). The role of hyaluronic acid in the design and functionalization of nanoparticles for the treatment of colorectal cancer. Carbohydr. Polym..

[31] Kim K.R., Kim H.Y., Lee Y.D., Ha J.S., Kang J.H., Jeong H., Bang D., Ko Y.T., Kim S., Lee H. (2016). Self-assembled mirror DNA nanostructures for tumor-specific delivery of anticancer drugs. J. Control. Release.

[32] Shi S., Chen Y., Tian T., Li S., Lin S., Zhang Y., Shao X., Zhang T., Lin Y., Cai X. (2020). Effects of tetrahedral framework nucleic acid/wogonin complexes on osteoarthritis. Bone Res..

[33] Wu T., Liu J., Liu M., Liu S., Zhao S., Tian R., Wei D., Liu Y., Zhao Y., Xiao H. (2019). A Nanobody-Conjugated DNA Nanoplatform for Targeted Platinum-Drug Delivery. Angew. Chem. Int. Ed. Engl..

[34] Brand D.D., Latham K.A., Rosloniec E.F. (2007). Collagen-induced arthritis. Nat. Protoc..

[35] Yoshikawa N., Yamada A., Yokota T., Moritake H., Hirabara Y., Ikeda R. (2021). Measurement of methotrexate in human cerebrospinal fluid using a chemiluminescence immunoassay intended for serum and plasma matrices. J. Clin. Lab. Anal..

[36] Bösmüller H., Pfefferle V., Bittar Z., Scheble V., Horger M., Sipos B., Fend F. (2018). Microvessel density and angiogenesis in primary hepatic malignancies: Differential expression of CD31 and VEGFR-2 in hepatocellular carcinoma and intrahepatic cholangiocarcinoma. Pathol. Res. Pract..

[37] Chistiakov D.A., Killingsworth M.C., Myasoedova V.A., Orekhov A.N., Bobryshev Y.V. (2017). CD68/macrosialin: Not just a histochemical marker. Lab. Investig..

[38] Kemble S., Croft A.P. (2021). Critical Role of Synovial Tissue-Resident Macrophage and Fibroblast Subsets in the Persistence of Joint Inflammation. Front. Immunol..

[39] Shymborska Y., Budkowski A., Raczkowska J., Donchak V., Melnyk Y., Vasiichuk V., Stetsyshyn Y. (2023). Switching it Up: The Promise of Stimuli-Responsive Polymer Systems in Biomedical Science. Chem. Rec..

