# Correction: Jin et al. A pH-Responsive DNA Tetrahedron/Methotrexate Drug Delivery System Used for Rheumatoid Arthritis Treatment. *J. Funct. Biomater*. 2023, *14*, 541

**DOI:** 10.3390/jfb15110349

**Published:** 2024-11-18

**Authors:** Yi Jin, Xingyu Ge, Yinjin Xu, Siyi Wang, Qian Lu, Aidong Deng, Jingjing Li, Zhifeng Gu

**Affiliations:** 1 Department of Rheumatology, Affiliated Hospital of Nantong University, Medical School of Nantong University, Nantong 226000, China; jin_yibai@126.com (Y.J.);; 2 Research Center of Clinical Medicine, Affiliated Hospital of Nantong University, Nantong 226000, China; 3 Department of Rheumatology, Yancheng Third People’s Hospital, Yancheng 224000, China; 15896260521@163.com; 4 Department of Hand Surgery, Affiliated Hospital of Nantong University, Nantong 226000, China

In the original publication [1], there was a mistake in Figure 4 as published. The two sub-images in Figure 4b-Liver are the same due to an error in the data uploading process. The corrected Figure 4 appears below. 

The authors state that the scientific conclusions are unaffected. This correction was approved by the Academic Editor. The original publication has also been updated.

## Figures and Tables

**Figure 4:**
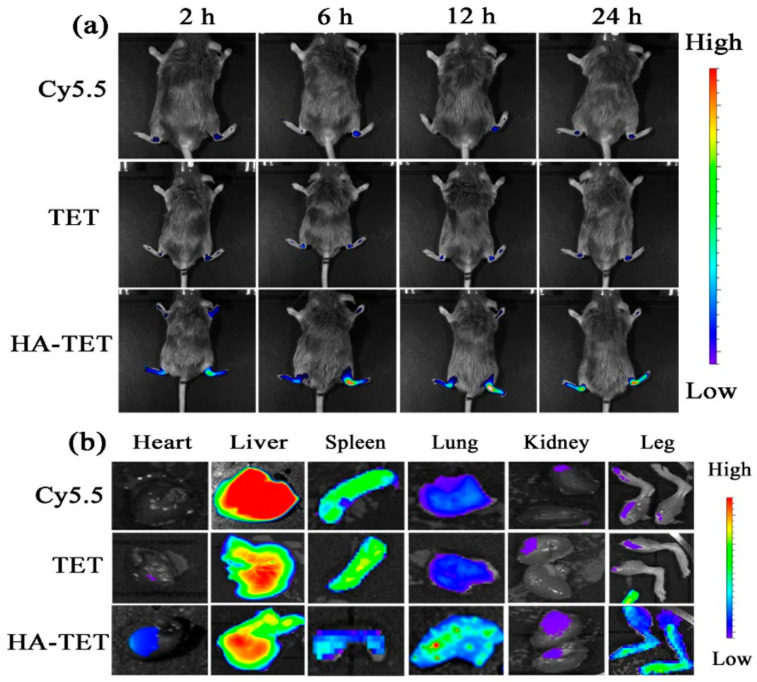
Living image of the CIA mice. (**a**) The overall view. (**b**) The image of each organ, such as the heart, liver, spleen, lung, kidney, and leg; (*n* = 3).
