# Supplementary material for: A pH-Responsive DNA Tetrahedron/Methotrexate Drug Delivery System Used for Rheumatoid Arthritis Treatment
Source: J Funct Biomater. 2023 Nov 3;14(11):541. doi: 10.3390/jfb14110541 (PMC10672632; doi:10.3390/jfb14110541)
Supplement: Supplementary file 1 [file jfb-14-00541-s001.zip › jfb-2626594-supplementary.pdf]

Electronic Supplementary Material

A pH-responsive DNA tetrahedron/methotrexate drug delivery system used for rheumatoid arthritis treatment

Yi Jin[1,2+], Xingyu Ge[3†], Yinjin Xu[1], Siyi Wang[1], Qian Lu[1], Aidong Deng[4], Jingjing Li[2,*], Zhifeng Gu[4,*]

[1] Department of Rheumatology, Affiliated Hospital of Nantong University, Medical School of Nantong University, Nantong, 226000, China; jin_yibai@126.com (Y.J.); 13862959217@163.com (Y.X.); siyiwang5@163.com (S.W.); luqian960927@163.com (Q.L.)

[2] Research Center of Clinical Medicine, Affiliated Hospital of Nantong University, Nantong, 226000, China

[3] Department of Rheumatology, Yancheng Third People's Hospital, Yancheng, 224000, China; 15896260521@163.com

[4] Department of Hand Surgery, Affiliated Hospital of Nantong University, Nantong, 226000, China; aidong_deng@163.com

*Correspondence: 13861903880@163.com (J.L.); guzf@ntu.edu.cn (Z.G.)

† These authors contributed equally to this work.

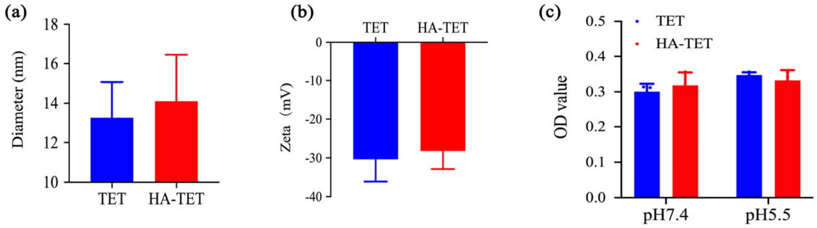

Figure S1. Characterization of HE-TET nanoparticles. (a) The particle size of TET and HA-TET. (b) The zeta potential of TET and HA-TET. (c) The OD value of TET and HA-TET in different pH.
